# The First Molecular Detection of the Presence and Prevalence of *Trypanosoma theileri* in Cattle from Türkiye

**DOI:** 10.1007/s11686-025-01059-2

**Published:** 2025-06-02

**Authors:** Omer Faruk Sahin, Ufuk Erol, Husnu Furkan Sakar, Kursat Altay

**Affiliations:** 1 Department of Parasitology, Faculty of Veterinary Medicine, Sivas Cumhuriyet University, Sivas, 58140 Türkiye; 2 Department of Parasitology, Institute of Health Sciences of Sivas Cumhuriyet, Sivas Cumhuriyet University, 58140 Sivas, Türkiye

**Keywords:** *Trypanosoma theileri*, First detection, Phylogeny, DNA sequencing, Cattle, Türkiye

## Abstract

**Purpose:**

*Trypanosoma theileri* is an opportunistic parasite that has worldwide distribution. This parasite has been detected in various hosts including cattle. Normally *T. theileri* can cause mild infection but it may lead to disease among animals in case of mixed infection or immunosuppression. In the present study, it was aimed to investigate the presence, prevalence, and to detect genotypes of *T. theileri* in cattle from different provinces in Türkiye using molecular techniques.

**Methods:**

In this study, 517 cattle blood collected from different provinces (Giresun, Samsun, Tokat, Sivas, Çorum, Trabzon, and Kastamonu) of Türkiye. Genomic DNA was extracted from blood samples using a commercial kit. The obtained gDNAs were screened for the presence of *T. theileri* using PCR. Partial parts of the *CATL* gene of randomly selected four positive samples were sequenced to determine the phylogenetic position of *T. theileri* Türkiye isolates.

**Results:**

PCR results showed that 18 out of 517 samples (3.48%) were positive in terms of *T. theileri*. The highest prevalence of *T. theileri* was observed in animals over 3 years of age (3.98%), followed by animals in the 1–3-year age group (3.16%). The prevalence of *T. theileri* was higher in female animals (3.62%) than in male animals (2.91%). The phylogenetic analyses of positive samples showed that *T. theileri* Türkiye isolates were clustered with the Tth IIB genotype of the *T. theileri* TthII lineage.

**Conclusion:**

In conclusion, the presence and prevalence of *T. theileri* in cattle were determined by molecular analyses for the first time in Türkiye. Moreover, Tth IIB genotype was also reported in Türkiye with this work. Although *T. theileri* is not thought to cause serious clinical symptoms in hosts, it should not be overlooked that it can cause significant economic losses in mixed infections with different pathogens.

## Introduction

Trypanosomes (Euglenozoa; Kinetoplastea; Trypanosomatida), which are among the most common and significant parasites worldwide, cause serious diseases in humans and animals [1]. *Trypanosoma theileri* is a hemoprotozoan classified in the subgenus *Megatrypanum*, which includes opportunistic species with low pathogenicity and high levels of genetic heterogeneity [2, 3]. *Trypanosoma theileri* is primarily transmitted by flies belonging to the Tabanidae family, but it is also spread by various species of ticks (*Hyalomma anatolicum* and *Rhipicephalus* (*Boophilus*) *microplus*) and keds [4–6]. In recent years, studies have reported that various species of mosquitoes belonging to the *Aedes* genus within the Culicidae family also serve as vectors to this parasite [7].

*Trypanosoma theileri* is an opportunistic parasite that is widespread worldwide. The pathogen has been detected in domestic and wild ruminants such as cattle, buffalo, deer, and sheep [7–12]. In recent years, *T*. *theileri* has also been detected in horses and bats [13, 14]. Parasitemia seen in hosts is usually characterized by nonspecific clinical symptoms. The pathogen may remain asymptomatic for a long time. However, high parasitemia may be seen in immunosuppressive conditions such as pregnancy, stress, various diseases (viral or microbial), and malnutrition [15, 16]. It has been reported that fever, loss of appetite, and anemia occur in some animals due to these conditions [16, 17].

*Trypanosoma theileri* can be diagnosed by microscopic examination, but in recent years it can also be detected by PCR-based molecular techniques [7, 12, 18, 19]. Molecular techniques are methods with higher specificity and sensitivity than other methods. In recent years, molecular-based studies based on DNA sequence analysis conducted on *internal transcribed spacer* (*ITS*), *18 S small subunit ribosomal RNA*, and *cathepsin L-like* (*CATL*) gene regions have reported the existence of two genetic lineages, named TthI and TthII, belonging to *T*. *theileri* [3, 15].

*Trypanosoma theileri* has been detected in many countries of the world with different climates and vector populations and has been identified in various hosts and vector species [18–23]. Türkiye has suitable climatic and geographical features for the viability of many vector species. Many vectors and vector-borne pathogens such as *Anaplasma capra*,* (A) phagocytophilum*,* Babesia bigemina*,* (B) bovis*,* B. divergens*,* Theileria annulata*,* Ehrlichia* spp., *Hepatozoon* spp., and *Mycoplasma* spp., have been identified in studies conducted in various part of Türkiye [24–38]. According to the literature review, no study has been found to date in Türkiye investigating *T*. *theileri* in cattle using molecular methods. This study aimed to determine the epidemiological presence of *T. theileri* in cattle by amplifying the *CATL* gene region, which is widely used in the detection of *T. theileri*, by PCR in cattle blood samples obtained from different provinces of Türkiye (Giresun, Samsun, Tokat, Sivas, Çorum, Trabzon, and Kastamonu) and to reveal the phylogenetic features of the species identified for the first time in Türkiye by DNA sequence analysis.

## Materials and methods

### Study Area and Samples

Located in the subtropical climate zone, Türkiye is located in the Eurasian continent, with part of its territory in the European continent and the other part in the Asian continent. This geographical structure of Türkiye adds richness and diversity to the country in terms of vegetation, while also creating a suitable environment for different vector species to live in the regions. In addition, this geographical feature plays a natural role in the transmission of intercontinental diseases [32].

**Fig. 1:**
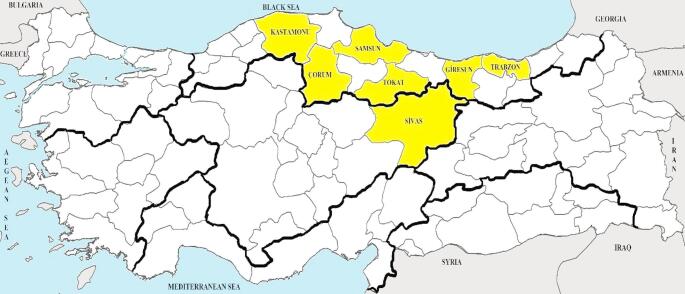
Location of sampling area in this study

Blood samples collected from cattle from seven different provinces (Giresun, Samsun, Tokat, Sivas, Çorum, Trabzon, and Kastamonu) of Türkiye were used in the study for other projects and stored in appropriate conditions in the research laboratory were used (Fig. 1). In the study, a total of 517 blood samples collected from cattle were investigated. The distribution of the investigated blood samples according to provinces, gender, and age is given in Table 1.

**Table 1:** The distribution of the examined blood samples by Province, gender, and age

Locations	Age (year)		Gender		Total
	1–3	> 3	Male	Female	
Giresun	36	34	7	63	70
Samsun	53	44	12	85	97
Tokat	65	35	29	71	100
Sivas	64	36	19	81	100
Çorum	35	15	23	27	50
Trabzon	28	22	8	42	50
Kastamonu	35	15	5	45	50
**Total**	**316**	**201**	**103**	**414**	**517**

### Genomic DNA Extraction from Blood Samples

Total genomic DNA (gDNA) was extracted from EDTA-treated blood samples using a PureLink™ Genomic DNA kit (Cat. No.: K1820–02, Invitrogen™, Carlsbad, USA). The extraction process was conducted in accordance with the manufacturer’s instructions. In order to prevent the occurrence of false positive results during the DNA isolation process, DNase-RNase-free sterile water (Cat. No. 129114, Qiagen^®^, Germany) was added to each DNA extraction group as a negative control. In addition, controls (Accession number: PP565860) that were previously found to be *T. theileri* positive in different studies were also used during extraction [12]. Genomic DNA, isolated from the subject, was eluted in a total of 200 µL of DNA elution buffer, supplied by the manufacturer, and stored at a temperature of -20 °C until further utilisation.

### PCR Assay for the Detection of *Trypanosoma theileri*

The gDNAs were investigated for *T. theileri* species with PCR assay using primers F (5’- CGTCTCTGGCTCCGGTCAAAC − 3’) and R (5’- TTAAAGCTTCCACGAGTTCTTGATGATCCAGTA − 3’) amplifying 289 bp of the *CATL* gene [39].

PCR assay was performed to a total volume 25 µL, including 10× PCR buffer (Invitrogen™, Carlsbad, USA), 2.5 µL MgCl_2_ (50 mM) (Invitrogen™, Carlsbad, USA), 200 µM of each dNTP (Cat.No.: R0181, Thermo Scientific™, Lithuania), 1 µL (10 pmol/µL) of each of the primers, 1.25 U of Taq DNA polymerase (5U/ µL) (Ref.No.: 100021276, Invitrogen™, Carlsbad, USA), 2.5 µL template DNA, and DNase-RNase-free sterile water.

The thermal cycling protocol used for PCR was 94 °C for 5 min, followed by 35 cycles of 94 °C for 1 min, 59 °C for 1 min, 72 °C for 1 min, and a final extension of 5 min at 72 °C. The resulting PCR products were loaded onto a 1% agarose gel and then electrophoresed at 90 volts for 60 min. Then, the agarose gel was stained with ethidium bromide for 20 min. The results were visualized with a UV transilluminator.

### Sequencing and Phylogenetic Analysis

Sequencing and phylogenetic analysis of the positive samples detected were performed. For this purpose, randomly selected positive samples that were representative study regions were sequenced with the primers used PCR assay. After the PCR products were visualized on the gel, gel purification was performed using a commercial gel extraction kit (PCR Clean-Up & Gel Extraction Kit, GeneDireX^®^, Cat.No.: NA006-0300). These samples were sent for sequence analysis by a commercial company (BM Labosis, Ankara). The sequence results were checked for chromatogram quality scores using the FinchTV (version 1.4.0) program (Geospiza Inc., Seattle, Washington, USA). Nucleotides with low chromatogram quality scores were trimmed. The consensus sequences were identified using MEGA-11 software [40]. The identified consensus sequences were uploaded to the GenBank system and then accession numbers were obtained.

Using MEGA-11 software [40], genetic diversity was evaluated between *T*. *theileri* identified in this study and other *T*. *theileri* species registered in GenBank. The maximum likelihood method was used to create phylogenetic trees. Before constructing the phylogenetic tree, it was determined that the best algorithm to be used in the phylogenetic tree of related pathogens was the Kimura-2 + G parameter model [41] using the Find Best-Fit Substitution Model in MEGA-11 and this algorithm was used in the phylogenetic tree. The bootstrap analysis (1,000 repetitions) was performed by 1,000 repetitions.

### Statistical Evaluation

Statistical analyses were performed using the chi-square test given age and sex parameters. *p* < 0.05 was accepted to be statistically significant.

### Ethics Statement

Ethical approval to conduct the study was obtained from the Sivas Cumhuriyet University Local Animal Experiments Ethics Committee (Approval number: 65202830–050.04.04–12).

## Results

**Fig. 2:**
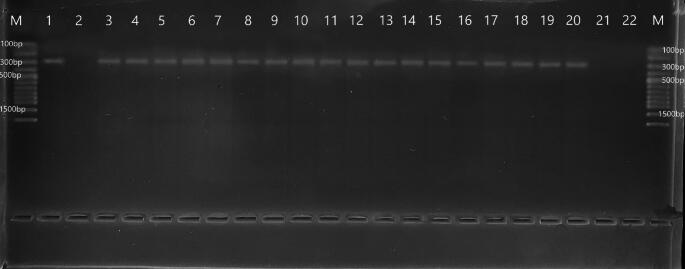
PCR products of *T. theileri* species. M. Marker, 1. *Trypanosoma theileri* positive control, 2. Negative control, 3–20. *Trypanosoma theileri* positive cattle samples, 21–22. Negative cattle samples

In this study, 517 cattle blood samples collected from different provinces were investigated by PCR analyses for *T*. *theileri.* According to PCR results, 18 of 517 (3.48%) samples were found to be infected with *T*. *theileri* (Fig. 2). In cattle older than 3 years, the prevalence of *T*. *theileri* was 3.98% (8/201), while this rate was 3.16% (10/316) in cattle aged 1–3 years. *T*. *theileri* was more prevalent in female (3.62%) than male (2.91%) (Table 2). As indicated by the results of the PCR, the distribution of cattle infected with *T*. *theileri* according to location, age, and gender is presented in the Table 2.

**Table 2:** PCR results for *Trypanosoma Theileri* according to location, age, and gender

Locations	Age						Gender						Total		
		1–3			> 3			Male			Female				
	*n*	+	%	*n*	+	%	*n*	+	%	*n*	+	%	*n*	+	%
Giresun	36	-	-	34	2	5.88	7	1	14.28	63	1	1.58	70	2	2.85
Samsun	53	-	-	44	1	2.27	12	-	-	85	1	1.17	97	1	1.03
Tokat	65	6	9.23	35	1	2.85	29	1	3.44	71	6	8.45	100	7	7
Sivas	64	4	6.25	36	4	11.11	19	1	5.26	81	7	8.64	100	8	8
Çorum	35	-	-	15	-	-	23	-	-	27	-	-	50	**-**	**-**
Trabzon	28	-	-	22	-	-	8	-	-	42	-	-	50	**-**	**-**
Kastamonu	35	-	-	15	-	-	5	-	-	45	-	-	50	**-**	**-**
**Total**	**316**	**10**	**3.16**	**201**	**8**	**3.98**	**103**	**3**	**2.91**	**414**	**15**	**3.62**	**517**	**18**	**3.48**

Randomly selected four positive samples, representative of the locations, were subjected to sequencing in order to confirm the results of the PCR. The consensus sequences obtained were then aligned with the *T*. *theileri* isolates from different countries that had been uploaded to GenBank. The accession numbers of *T*. *theileri* detected in cattle in the study are as follows: PV111771-PV111774. Our sequences exhibited 100% identically with each other. It was found that there was a high nucleotide similarity (93.28–100%) between our *T. theileri* isolates and *T. theileri* isolates uploaded to GenBank from different parts of the world. *Trypanosoma theileri* sequences obtained in this study had 100% nucleotide similarity to those of *T*. *theileri* identified in Sri Lanka (AB930159, cattle), Vietnam (LC125455, buffalo), Ecuador (ON063530, cattle), Kyrgyzstan (PP565860, cattle), Brazil (GU299363, KU587669 cattle), Colombia (MN718440, bat), and Japan (LC385970, LC385953, cattle). Moreover, our sequences had high nucleotide similarities with Vietnam (99.58%, LC125453, buffalo), Sri Lanka (99.58%, AB930158, cattle), Brazil (99.55%, GU299351, cattle), Colombia (99.52%, MN718442, bat), Japan (99.51%, LC385963, cattle), Vietnam (99.16%, LC125454, cattle), Japan (99.02%, LC385982, cattle), Japan (98.05%, LC618042, sika deer), and Sri Lanka (93.28%, AB930161, buffalo) isolates.

**Fig. 3:**
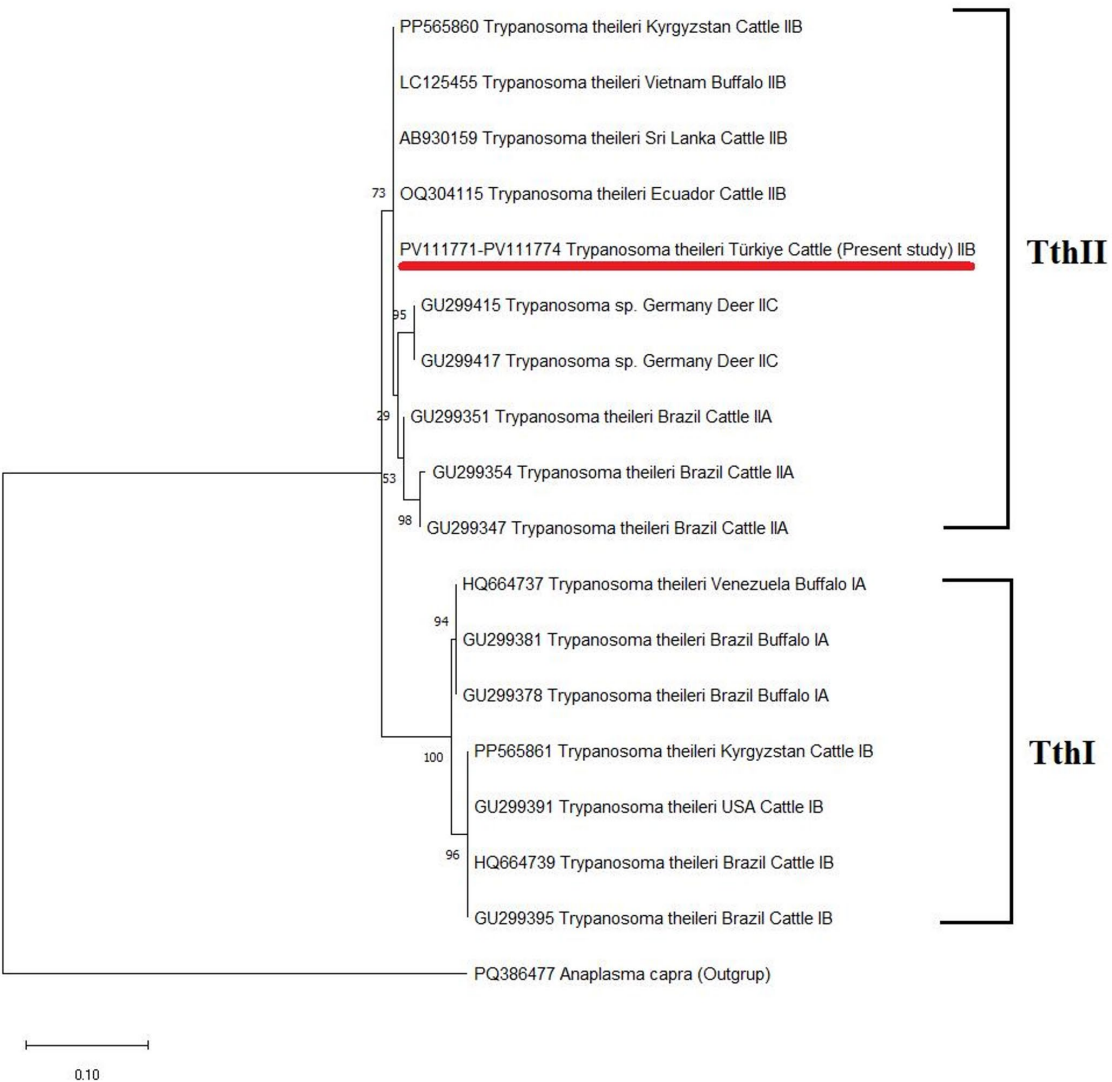
Phylogenetic tree of *T. theileri CATL* gene sequence. *Trypanosoma theileri* TthII (IIB) isolates identified in the study are underlined. Numbers at the nodes represent the bootstrap values with 1,000 replicates. The evolutionary history was inferred by using the Maximum Likelihood method and the Kimura-2 + G parameter model [41]. The scale bar represents 0.10 substitutions per nucleotide position. Evolutionary analyses were performed in MEGA-11 [40]

Phylogenetic analysis of the *CATL* gene was performed by aligning our *T. theileri* sequences with different *T. theileri* isolates present in GenBank. The phylogenetic tree shows that the *T. theileri* species obtained in this study belong to the same lineage (TthII) and cluster with the same *T. theileri* species described from different countries (Fig. 3). At the same time, as a result of phylogenetic analysis, it was determined that the genotypes identified were Tth IIB genotype of *T. theileri* TthII lineage.

## Discussion

Vectors and vector-borne pathogens threaten animal and human health in almost all regions of the world. In recent years, the importance of vector-borne pathogens has increased due to several reasons such as global warming and human movements [42, 43]. Infections and deaths caused by vectors and vector-borne pathogens cause billions of dollars in economic losses every year [42]. Trypanosomiasis in animals, especially livestock, is a major obstacle to livestock development in some rural areas of the world affected by the disease. In the studies carried out, the disease has been detected in various hosts (such as cattle, buffalo, deer, sheep) [7, 9–11]. This parasite has been reported in a variety of blood-sucking arthropods, including tabanids, mosquitoes, deer keds, tsetse flies, and tick species [7, 44]. There are only limited data available on the prevalence and distribution of *T. theileri* in comparison to other vector-borne diseases.

Türkiye is located in a subtropical climate zone where many different vector species live. For this reason, many studies have been performed to investigate the epidemiology, prevalence and distribution of vector-borne pathogens in Türkiye and various vector-borne pathogens have been detected in the country, especially among domestic animals [24–38]. According to the literature review, there are no molecular studies on *T. theileri* species in cattle in Türkiye. In this study, *T*. *theileri* species were investigated by molecular analyses for the first time in cattle blood collected from different climatic regions of Türkiye. In addition, phylogenetic analyses were performed by sequence analyses from the positive species.

*Trypanosoma theileri* has been detected in cattle in molecular studies conducted in different parts of the world [45–48]. In these studies, *T. theileri* has been reported in Ecuador (15.6%) [46], Philippines (12-15.7%) [47, 49], Japan (32.9%) [45], Sri Lanka (%7.6) [8], Ecuadorian Amazon (11.4%) [50], Brazil (8.13%) [51], Thailand (20.6%) [48], and Venezuela (5%) [52]. Although microscopic detection of *T. theileri* was performed in Türkiye by Inci and Sayın [53], there is no molecular study on the detection of *T. theileri* to date. In this study, *T. theileri* was detected in 3.48% of cattle blood samples obtained from different regions of Türkiye using PCR assay. Our prevalence value was lower than all studies presented above. The prevalence of vector-borne pathogens may vary depending on various factors such as climatic characteristics of sampling regions, presence and abundance of vector species in sampling regions, number of animals researched in the studies, management of animals (barn or graze) researched, and sensitivity and specificity of methods used [45–47, 49, 50, 54, 55].

Studies revealed that age and sex factors may change the process of *T*. *theileri* infections in cattle [48, 56]. In this study, it was found that *T. theileri* infections in cattle over 3 years of age (3.98%) were higher than in cattle under 3 years of age (3.16%). However, this result was not statistically significant (*p* > 0.005). In studies similar to our study, it was found that older cattle were more infected than younger cattle. This situation is explained by reasons such as increased frequency of vector contact and maintenance of maternal antibody levels in young animals [48]. Moreover, *T. theileri* was more prevalent in female cattle (3.62%) compared to male cattle (2.91%) in the study. These results were not statistically significant on gender (*p* > 0.005). Arnuphapprasert et al. [48] reported that *T*. *theileri* positive male cattle were more common than females. Similarly, in the study by Chávez-Larrea et al. [46] a higher prevalence was found in males compared to females. *Trypanosoma theileri* is known to be a vector-borne protozoon. In this study, the prevalence was found to be higher in female cattle older than three years. This may be related to the fact that older animals are exposed to ectoparasites more than younger animals [46, 48]. In addition, female cattle go out to pasture more than male cattle in Türkiye. In this case, it is thought that females are exposed to vectors more than males and the pathogen is more common in females.

Studies have reported that *T*. *theileri* is characterized by various lineages and genotypes in different hosts (such as cattle, buffalo, antelope, bat, and horse) [9, 12–14, 47, 55]. Molecular and DNA sequence analysis were used to determine the presence of *T. theileri* genotypes and two *T. theileri* isolates (TthI and TthII) are circulating in cattle herds [12, 45, 51]. Within these lineages, several sub-genotypes have been identified as being specific to each host species, as is the case for cattle (IIA, IB, IIB, IC), buffalo (IA), and deer (IIC) [3, 10, 55]. In this study, the DNA sequence of the *CATL* gene was obtained from four positive samples selected to represent the regions to determine the *T. theileri* isolates in the country and it was determined that the IIB genotype in the TthII lineage is circulating among cattle in Türkiye. It is seen that the results of the study are compatible with other results in cattle in the world [46, 50, 55].

## Conclusion

In this study, *T. theileri* was identified in cattle for the first time in Türkiye using molecular and phylogenetic analysis. The prevalence of *T. theileri* in cattle was found to be 3.48% in different provinces of Türkiye. In addition, phylogenetic analyses revealed that the species belong to genotype IIB. The present study has shown that *T. theileri* species are circulating in cattle in Türkiye. Although *T. theileri* infections are known to be low-pathogenic, extended studies are needed to evaluate the effects on cattle and other hosts when evaluated together with other infectious agents in the region.

## Data Availability

No datasets were generated or analysed during the current study.
